# HaloClass: Salt-Tolerant Protein Classification with Protein Language Models

**DOI:** 10.1007/s10930-024-10236-7

**Published:** 2024-10-21

**Authors:** Kush Narang, Abhigyan Nath, William Hemstrom, Simon K. S. Chu

**Affiliations:** 1 College of Biological Sciences, University of California, Davis, USA; 2 Department of Biochemistry, Pt. Jawahar Lal Nehru Memorial Medical College, Raipur, India; 3 Department of Biological Sciences, Purdue University, West Lafayette, USA; 4 Biophysics Graduate Program, University of California, Davis, USA

**Keywords:** Protein classification, Protein salt tolerance, Halophilic proteins, Protein language models

## Abstract

Salt-tolerant proteins, also known as halophilic proteins, have unique adaptations to function in high-salinity environments. These proteins have naturally evolved in extremophilic organisms, and more recently, are being increasingly applied as enzymes in industrial processes. Due to an abundance of salt-tolerant sequences and a simultaneous lack of experimental structures, most computational methods to predict stability are sequence-based only. These approaches, however, are hindered by a lack of structural understanding of these proteins. Here, we present HaloClass, an SVM classifier that leverages ESM-2 protein language model embeddings to accurately identify salt-tolerant proteins. On a newer and larger test dataset, HaloClass outperforms existing approaches when predicting the stability of never-before-seen proteins that are distal to its training set. Finally, on a mutation study that evaluated changes in salt tolerance based on single- and multiple-point mutants, HaloClass outperforms existing approaches, suggesting applications in the guided design of salt-tolerant enzymes.

**Supplementary Information:**

The online version contains supplementary material available at 10.1007/s10930-024-10236-7.

## Introduction

Improvements in protein sequencing technology have facilitated massive advances in the understanding of salt-tolerant proteins [1]. Characterizing salt-tolerant proteins is important for industrial processes, pharmaceuticals, and microbiology [2–4]. However, despite an abundance of sequences, there remains a limited number of experimental salt-tolerant protein structures (5), limiting biologists from conducting more in-depth structural analysis.

As a consequence of this limitation, many existing computational approaches to salt-tolerant protein classification are designed to be sequence-based only. These methods all leverage human-interpretable features, manually selected by experts in the field. The classifier created by Zhang et al. [6] relies on only amino acid frequencies for protein characterization. Although this approach is intuitive, it loses essential information encoded in a protein’s primary sequence, most notably, the proximities and interactions between neighboring and distant amino acids. The model introduced by Nath [7] improves upon Zhang’s model by adding dipeptide counts, isoelectric points, and other human-interpretable physicochemical properties. More recently, Hu et al. [8] published an approach that leverages ensemble learning. However, none of these approaches use structural knowledge about secondary and tertiary structure.

The recent development of protein language models (pLMs) has been an important advancement in computational and structural biology [9]. pLMs, such as ESM-2, learn to predict the identity of masked amino acids using the context provided by the rest of the protein sequence [10]. Through this process, pLMs learn to derive and predict the secondary and tertiary structure of novel proteins, using only evolutionary information from amino acid sequences [10–12]. By avoiding sequence alignment, pLM-based structure predictors have significantly faster runtimes and lower computational demand than alignment-dependent methods, such as AlphaFold [13, 14] or RosettaFold [15]. Using pLM representations for classification is an incredibly versatile approach that has achieved state-of-the-art performance in domains ranging from thermophilicity prediction [16] to ion channel identification [17] to small molecule binding site prediction [18].

In light of these successes, we present HaloClass, an SVM classifier that leverages ESM-2 representations for protein sequences. Trained on a larger and more diverse dataset than its predecessors, HaloClass establishes a new state-of-the-art for salt tolerance classification and generalizes better than existing models. HaloClass successfully differentiates between proteins from salt-tolerant and non-tolerant organisms, from structurally similar homologs, and from structurally identical mutants. HaloClass is open-source and available on GitHub and Google Colab.

## Materials and Methods

### Datasets

We used three datasets to train HaloClass and test all the other classifiers:

1. **The Zhang dataset**: Zhang et al. [6] published a dataset for halophilic protein classification by collecting 139 sequences from salt-tolerant *Salinibacter ruber DSM 13,855* and another 139 from non-tolerant *Pelodictyo luteolum* DSM 2379. The original set of 278 sequences were used to train Zhang’s and Nath’s classifiers.
2. **The Siglioccolo dataset**: Siglioccolo et al. [19] created a dataset with 15 pairs of homologous salt-tolerant and non-tolerant proteins with experimental structures. We selected only the 8 pairs that contained a salt-tolerant protein from an organism bearing salt tolerance adaptations. The remaining 7 pairs were excluded, since their salt-tolerant proteins came from organisms that achieved halophilicity through osmotic pressure. As such, these proteins would not be expected to bear salt-tolerant adaptations.
3. **The new datasets**: We used 38,361 sequences from 14 different organisms, accessed from UniProt [20]. These 14 organisms were selected to enhance HaloClass’s generalizability. Among the 5 salt-tolerant organisms, the HaloDom database annotated 3 as Moderate and 2 as Extreme halophiles [21]. The negative set includes organisms that are mesophiles, thermophiles, and acidophiles to ensure that HaloClass effectively differentiates salt-tolerance from other extremophile adaptations. Both sets include both archaea and bacteria species. We applied a CD-HIT [22] cutoff of 50% to avoid overfitting on specific types of proteins and to reduce overlap between training and testing datasets to better assess generalizability. After clustering, we were left with 28,030 sequences. These sequences were split into three groups: training, evaluation, and testing, in a 90-5-5 split. The specific organisms and sequence counts are tabulated in Supplementary Table 1. FASTA files with all the sequences are available in the supplementary material.

### Existing Models

We evaluated two existing classifiers on our datasets:

1. **Zhang’s model**: Zhang et al. [6] considered several classification approaches. They reported their simple linear regression model achieved the highest test set accuracy. We reimplemented this approach by implementing the linear equation in Python with coefficients up to three decimal points, as listed in the original publication.
2. **Nath’s model**: Nath [7] evaluated fourteen different machine learning approaches, all applied to the same 454-long feature vector derived with manually picked features. These features present include fractions of all combinations of dipeptides, average physicochemical properties (e.g. residue bulkiness and flexibility) and isoelectric point. We evaluated their highest performing model in Weka.

### HaloClass

HaloClass is an SVM classifier trained on features extracted from ESM-2, a protein masked language model [10, 11]. For all cases, ESM-2 representations were extracted from the last hidden layer of the ESM-2 model. Representations were generated in batches of 32, padded to the max-length and truncated to a maximum of 1022 tokens, including a start and end of sequence token. Subsequently, for each sequence, representations for only the amino acid tokens were selected and mean-pooled across the sequence dimension. This reduced the representation size to Nx1 for all test cases, where N refers to the hidden layer size of the respective ESM-2 checkpoint (for instance, the ESM-2 150 M checkpoint has a *N* = 640). Performance metrics for different classification models trained on the 35 million parameter ESM-2 checkpoint are shown in Supplementary Fig. 1. We selected an SVM architecture due to its performance and relative model simplicity compared to other options. ESM-2 is available in several checkpoints, each with hidden-layer representations with different dimensions. Performance metrics for SVM models trained on the different ESM-2 checkpoints are shown in Supplementary Fig. 2. We selected the 150 million ESM-2 checkpoint for representations due to lower runtime and storage costs than the 650 million checkpoint. Lastly, we swept through the SVM kernel and hyperparameters, as specified in Supplementary Figs. 3 and 4. Because all kernels performed similarly, we selected the linear kernel to reduce training time and selected the best-performing hyperparameters C = 0.1 and gamma = 10^− 6^. For all subsequent analysis, HaloClass refers to the SVM model with a linear kernel and default parameters, trained on the 150 million parameter ESM-2 checkpoint.

### Mutation Study

**Fig. 1:**
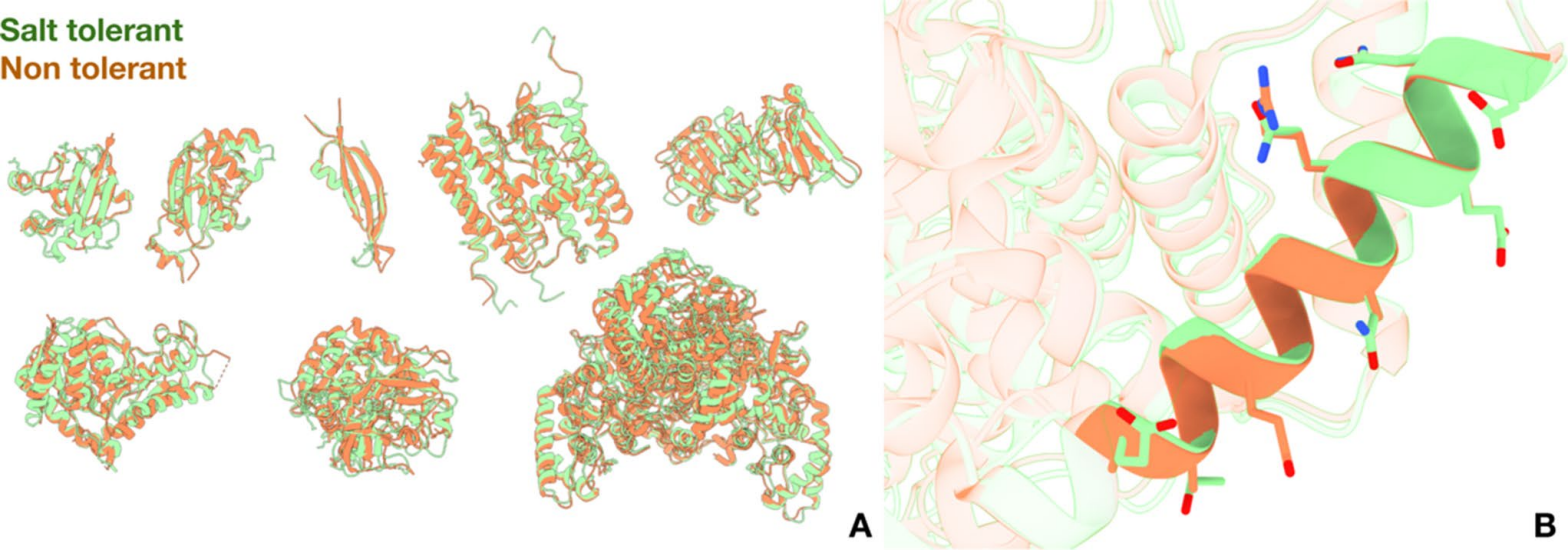
Structural comparison of homologous salt-tolerant and non-tolerant proteins. Salt-tolerant structures are shown in green and non-tolerant structures are in orange. In panel **A**, all eight homologous pairs analyzed from Siglioccolo et al. [19] are superimposed. In panel **B**, a helix from two catalase-peroxidases is highlighted (salt-tolerant: 1ITK; non-tolerant: 2FXG)

**Fig. 2:**
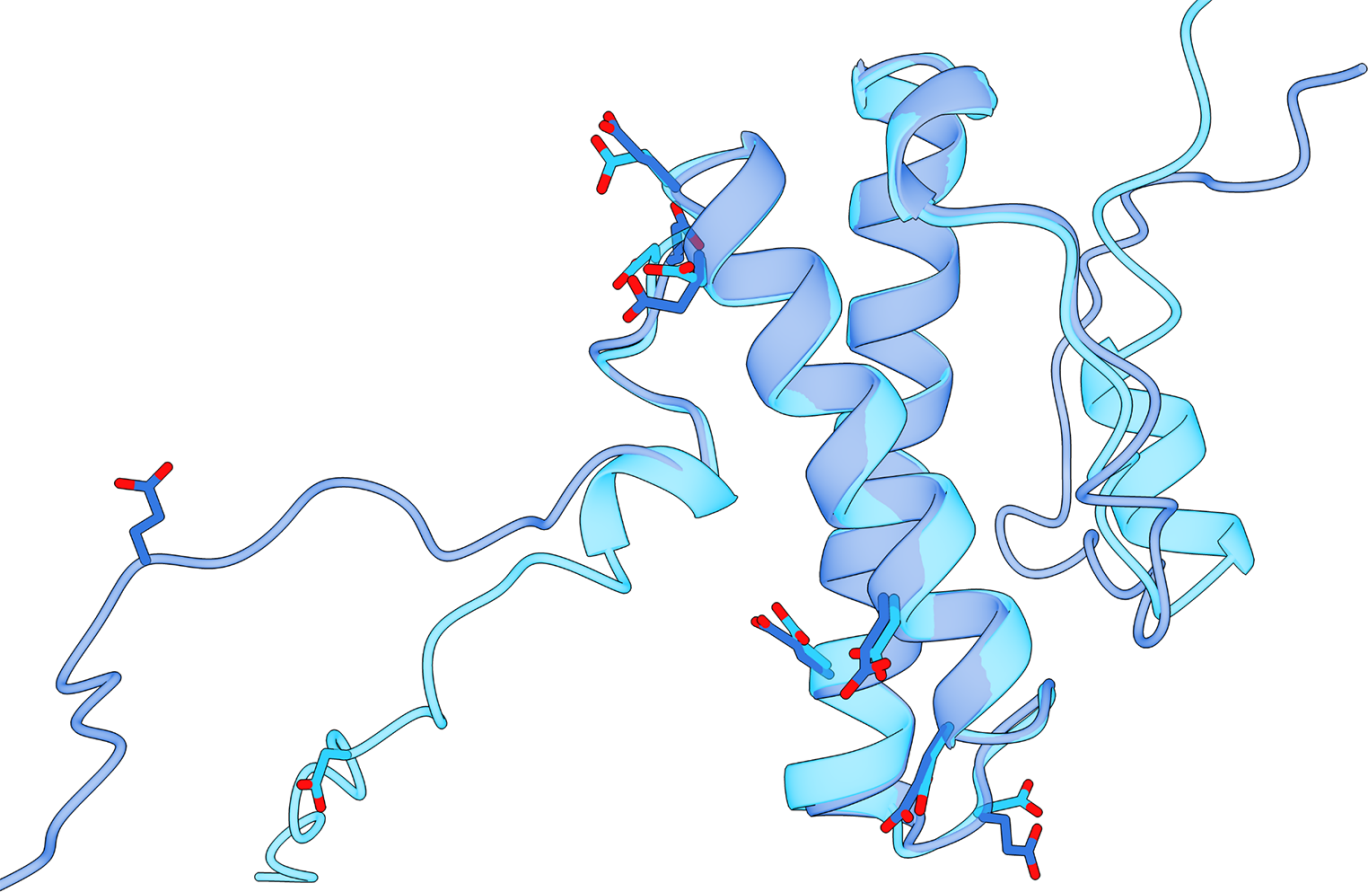
Models for the wild-type and a mutant of the salt-tolerant DNA ligase N. The figure shows superimposed AlphaFold 3 structures for the DNA ligase N from Haloferax volcanii. The wild-type is in light blue, and the mutant type with 8 aspartic acids mutated to glutamic acids is in dark blue; mutated side-chains are displayed. The structures have an RMSD of 0.62Å

We performed a comparative mutation study with HaloClass and Nath’s model to assess their ability to assist in guided protein design. Tadeo et al. [23] reported the experimental salt tolerance of more than one hundred mutants of 3 wild-type proteins. Many of these mutants were not analyzed, lacked definitive effects, or had other complications. We employed the following process to select which specific mutants to computationally test:

1. We only selected mutants marked as expressed and tested in the Supplementary Tables.
2. We used Figs. 1 and 2 to determine the predicted salt tolerance based on two different metrics described in Tadeo:
  - For cases where the two metrics disagreed about predicted salt tolerance, we excluded the protein.
  - For cases only one metric was reported, we used that to determine experimental salt tolerance.
  - For cases where neither metric was reported, we excluded the protein from analysis.
3. Based on the Figures, in cases where the error bar showed ambiguity between increases and decreases in salt tolerance, we excluded the protein.
  - a. We also excluded cases where the marker was so close to the baseline that it would obscure any error bars.
4. If a protein was successfully analyzed and tested via two pathways, we only considered the one that was listed without an asterisk.

This process left us with 49 mutants for 3 wild-type proteins that we used as the Tadeo dataset. The experimental changes in salt tolerance for these mutants (versus their wild-types) were compared against predicted changes by HaloClass and Nath’s model to evaluate accuracy.

### Structural Visualization

All structures are visualized in UCSF ChimeraX [24]. The AlphaFold 3 server was used to generate structural models for Fig. 2, all on default settings [13]. The proteins were superimposed using the ChimeraX matchmaker tool, and side-chains on the eight mutations between aspartic acid and glutamic acid are shown. In all other figures, structures are accessed from RCSB Protein Data Bank [25].

## Results

HaloClass is an SVM classifier trained on sequence embeddings from ESM-2. The details for the model architecture and training process are discussed in the Methods section. To effectively evaluate the discriminative power of HaloClass versus other state-of-the-art classifiers, we theorized a three-level evaluation system that tests algorithms on their abilities at the organism level, structure level, and mutation level.

### Evaluating at the Organism Level

Despite the abundance of salt-tolerant sequences, resolved three-dimensional structures of these sequences remain scarce. Sequence-based approaches circumvent this limitation by avoiding the need for modeled or experimental structures. We benchmarked three models on two datasets containing sequences annotated by the salt tolerance of the source organism. The Zhang dataset consists of 278 sequences, half from salt-tolerant *Salinibacter ruber* and the other half from non-tolerant *Pelodictyo luteolum* [6]. Here, HaloClass scores an accuracy of 94%, compared to 86% for Nath’s model and 63% for Zhang’s model. Similarly, with an AUROC of 0.99 and an MCC of 0.88, HaloClass outperforms the other approaches (See Table 1).

**Table 1:** Performance of state-of-the-art models on three benchmark datasets area under the receiver operating characteristic curve (AUROC) is a measurement where a perfect score of 1.0 indicates that every salt-tolerant protein was classified as more salt-tolerant than every non-tolerant protein. Zhang’s model does not provide confidence values for predictions, and therefore, does not have AUROC metrics or mutation level performance reported. Accuracy for the Tadeo dataset refers to the model’s accuracy at predicting the directionality of changes in salt tolerance (i.e. increase or decrease)

	Organism level						Structure level			Mutation level
	Zhang dataset			New test set			Siglioccolo dataset			Tadeo dataset
	Accuracy	AUROC	MCC	Accuracy	AUROC	MCC	Accuracy	AUROC	MCC	Accuracy*
Nath model	0.86	0.86	0.72	0.80	0.79	0.58	**0.94**	0.94	**0.88**	0.41
Zhang model	0.63	-	0.27	0.65	-	0.23	0.69	-	0.38	-
HaloClass	**0.94**	**0.99**	**0.88**	**0.98**	**0.99**	**0.96**	**0.94**	**1.00**	**0.88**	**0.94**

Interestingly, both Zhang’s and Nath’s models were trained on the Zhang dataset, meaning that those approaches have already seen these particular sequences. In other words, with these results, we show that HaloClass was able to generalize better to sequences absent in both training and validation sets than old approaches were able to understand data they already learned from.

We were interested in more rigorously evaluating the algorithms’ ability to generalize to new data, so we created a new test set with 1,402 sequences from 14 organisms. The sequences for this dataset were clustered to 50% sequence identity to minimize information leakage across dataset split, and yet still share similar sequence identities to Nath’s and Zhang’s training data (Supplementary Table 2). We believe it is a fair comparison of the generalizability of each approach. On our new test set, HaloClass scores an accuracy of 98% and an AUROC of 0.99, compared to 80% and 0.79 for Nath’s model, respectively. Here, HaloClass makes fewer than 30 classification errors on a dataset of more than 1,400 sequences. More performance metrics for HaloClass, specifically precision, recall, and F1 score, are presented in Supplementary Table 3.

### Evaluating at the Structure Level

Next, we evaluated these models on the Siglioccolo dataset [19] from which we selected 8 pairs of homologous protein structures from 12 different organisms. The root-mean-square deviation (RMSD) between each homologous structural pair ranges from 0.7Å to 1.2Å, with an average sequence similarity of 46.1% (Table 2). Supplementary Table 4 provides more information about the dataset. For evaluation on a paired dataset, classification errors and ranking errors are both important metrics. We define a ranking error as any salt-tolerant protein being assigned a lower confidence of being salt-tolerant than any other non-tolerant protein.

**Table 2:** Homologous structure pairs from Siglioccolo dataset and their similarity sixteen proteins were selected from Siglioccolo et al. [19] with pairwise sequence identity and RMSD reported between the salt-tolerant and non-tolerant homologs. 4-digit PDB codes are provided for included structures

Type	Salt-tolerant PDB	Not salt-tolerant PDB	Identity	RMSD (Å)
Ferredoxin	1DOI [26]	1FXA [27]	51.4%	0.8
DNA-protecting protein	1TJO [28]	2VXX [29]	36.6%	0.7
Glucose dehydrogenase	2B5W [30]	2CD9 [31]	50.0%	1.2
Dodecin	2CC6 [32]	2V18 [33]	42.2%	0.7
Catalase-peroxidase	1ITK [34]	2FXG [35]	60.9%	0.8
Nucleoside diphosphate kinase	2AZ3 [36]	3B54 [37]	54.2%	0.7
Malate dehydrogenase	2J5K [38]	1Y6J [39]	37.5%	1.2
Proliferating cell nuclear antigen	3IFV [40]	1RWZ [41]	36.3%	1.2

Nath’s model and HaloClass both make a single classification error on this dataset. However, with a perfect AUROC, HaloClass makes no ranking errors, compared to one ranking error made by Nath’s model. Additionally, the Siglioccolo dataset served as the evaluation dataset in the design of Nath’s model, thereby providing it an advantage on this dataset. In contrast, HaloClass was designed without knowledge of the Siglioccolo dataset, making it serve as a more independent test set.

As a comparative analysis, we superimposed these homologous structural pairs in Fig. 1A. In Fig. 1B, we highlight the differing surface residues on an alpha helix from a pair of catalase-peroxidases [salt-tolerant: 1ITK [34]; non-tolerant: 2FXG [35]]. This helix bears mutations at 8 positions. In comparison to the non-tolerant protein, 7 sites in the salt-tolerant helix increase their charge upon mutation. Broadly, this trend reflects the biophysical understanding that increased surface charge contributes to greater protein stability [42] and specifically for salt-tolerant proteins [43].

More specifically, G509 in the non-tolerant protein mutated to glutamic acid, E526, on the salt-tolerant homolog. This mutation is consistent with past work showing that surface glutamic acids increase protein solubility [44] and decrease aggregation [45]. Three alanines in the non-tolerant helix mutated to glutamic acid, threonine, and asparagine, respectively. These substitutions are consistent with the analysis from Nath [7] finding increased glutamic acid and threonine composition in salt-tolerant proteins. Past work has also demonstrated mutations away from alanine being linked to increased salt tolerance in a *Sorghum* crop [46].

### Evaluating at the Mutation Level

An important achievement for a protein classifier is the ability to assist in protein engineering tasks by predicting changes in salt tolerance in-silico, saving time and money by screening prior to experimental testing. As such, we are interested in evaluating HaloClass’s ability to predict changes in salt tolerance for single- and multiple-point mutations. Experimental data from Tadeo et al. [23] evaluated the salt tolerance for 49 of mutants of 3 wild-type proteins. These mutants range from 1 to 9 sites and span across a variety of protein classes and organisms, including 1 A domain of NAD^+^-dependent DNA ligase N from *Haloferax volcanii*, 1 A domain of NAD^+^-dependent DNA ligase N from *Escherichia coli*, and the IgG binding domain of protein L from *Streptococcus magnus*. Our findings are presented in Table 3.

**Table 3:** Predicted changes in salt tolerance for mutants compared to experimental data 49 mutants of 3 wild-type proteins from Tadeo et al. [23] are listed. Each mutant is described with its changes on side-chain identity, charge, and length as well as the number of sites mutated. Experimental results are presented alongside predictions from HaloClass and Nath’s model. No predicted change for Nath’s model means the prediction confidence was identical to the wild type up to 4 decimal places

Protein	Mutation changes on				Experimental	Prediction	
	Charge	Length	Substitution	Sites		HaloClass	Nath
Protein L	none	-	E to D	3	+	+	none
Protein L	none	-	E to D	4	+	+	none
Protein L	none	-	E to D	6	+	+	none
Protein L	none	+	DN to EQ	2	+	-	none
Protein L	none	+	DN to EQ	3	-	+	none
Protein L	none	+	DN to EQ	4	-	-	none
Protein L	Neutral to negative	none	NQ to DE	3	+	+	+
Protein L	Neutral to negative	none	NQ to DE	4	+	+	+
Protein L	Neutral to negative	none	NQ to DE	5	+	+	-
Protein L	Negative to neutral	none	E to Q	3	+	+	-
Protein L	Negative to neutral	none	E to Q	5	+	+	-
Protein L	none	+	K to R	3	+	+	-
Protein L	none	+	K to R	5	+	+	-
Protein L	none	+	K to R	7	+	+	+
Protein L	Positive to neutral	-	K to S	1	+	+	+
Protein L	Positive to neutral	-	K to S	3	+	+	+
Protein L	Positive to neutral	-	K to S	5	+	+	+
Protein L	Positive to negative	-	K to E	2	+	+	+
Protein L	Positive to negative	-	K to E	3	+	+	+
Protein L	Positive to negative	-	K to E	4	+	+	+
Protein L	Positive to negative	-	K to E	5	+	+	+
Protein L	Positive to negative	-	K to E	6	+	+	+
Protein L	Positive to negative	-	K to E	7	+	+	+
Protein L	Positive to neutral	-	K to Q	2	+	+	+
Protein L	Positive to neutral	-	K to Q	3	+	+	+
Protein L	Positive to neutral	-	K to Q	4	+	+	+
Protein L	Positive to neutral	-	K to Q	5	+	+	+
Protein L	Positive to neutral	-	K to Q	6	+	+	+
Protein L	Positive to neutral	-	K to Q	7	+	+	-
Protein L	Negative to positive	+	DE to K	7	-	-	-
Protein L	Negative to positive	+	DE to K	8	-	-	+
Halo DNA ligase N	none	-	E to D	2	+	+	none
Halo DNA ligase N	none	-	E to D	6	+	+	none
Halo DNA ligase N	none	-	E to D	9	+	+	none
Halo DNA ligase N	none	+	D to E	4	-	-	none
Halo DNA ligase N	none	+	D to E	7	-	-	none
Halo DNA ligase N	none	+	D to E	8	-	-	none
Halo DNA ligase N	Negative to neutral	none	DE to NQ	7	-	-	-
Halo DNA ligase N	none	-	R to K	2	-	-	none
Halo DNA ligase N	none	-	R to K	3	-	-	none
Halo DNA ligase N	none	-	S to K	4	-	-	-
E. coli DNA ligase N	none	-	E to D	1	+	+	none
E. coli DNA ligase N	none	-	E to D	2	+	+	none
E. coli DNA ligase N	none	-	E to D	3	+	+	none
E. coli DNA ligase N	none	-	E to D	4	+	+	none
E. coli DNA ligase N	none	-	E to D	5	+	+	none
E. coli DNA ligase N	none	+	D to E	3	+	-	none
E. coli DNA ligase N	none	+	D to E	4	-	-	none
E. coli DNA ligase N	Neutral to negative	none	Q to E	2	+	+	-

The mutants surveyed differ in whether they altered only a side-chain’s charge, only length, or both simultaneously on the side-chain. Existing biophysical knowledge suggests that if the length of a side-chain is held constant, charge correlates with stability. Of the 7 mutants with unchanged lengths, HaloClass makes no errors. Here, Nath’s model struggles, accurately predicting just 3 of the 7 mutants. One possible explanation is provided by past work suggesting that the stabilizing effects of surface charges are not just dependent on net charge, but instead are influenced by long-distance charge-charge interactions in the unfolded state [47]. Nath’s interpretable representations have no ability to encode long-distance interactions like these by including only a portion of the primary sequence. In contrast, ESM-2 has been shown to learn long-distance residue-residue contacts [12] and has been used to assist for the protein structure prediction [10].

Simultaneously, it is generally expected that stability correlates with shorter residues, as longer side-chains are known to interfere with favorable solvent interactions [5, 7, 23]. Salt-tolerant proteins have generally evolved to bear fewer bulky residues [48]. There were 25 mutants where residue charge was held constant among which HaloClass made 3 classification errors with an accuracy of 88%. We believe this suggests that *HaloClass has a slightly lower resolution at the impact of length than charge upon mutation*.

Interestingly, *all 3 of HaloClass’s mistakes are attributable to changes between glutamic acids and aspartic acids*. One possible explanation is provided by a molecular dynamics study from Lemke et al. [49] which found unique differences in the ionic interactions of aspartic acid and glutamic acid oligomers, suggesting a more complex relationship than conventionally theorized. It is possible that our pLMs are unable to properly capture this nuance. Moreover, both Fukuchi et al. [50] and Nath [7] found a compositional preference for surface aspartic acids over glutamic acids in salt-tolerant proteins. In fact, Fukuchi et al. [50] hypothesizes that increases in aspartic acid might be the only evolutionary significant difference in amino acid composition between salt-tolerant and non-tolerant proteins and that any other compositional changes are side-effects of the aspartic acid trend.

Among the 17 mutations that simultaneously adjusted both side-chain length and charge, HaloClass accurately classified them all with an accuracy of 100%. This performance is surprising due to the competing influences of these mutations. Examining further, experimental results found that all arginine-to-lysine substitution increased salt tolerance and all removals decreased salt tolerance. HaloClass accurately predicted these experimental outcomes in all 5 cases. Past work shows that arginines, which can form up to 5 hydrogen bonds, are uniquely stabilizing compared to lysines, especially in the environment of lipids and membranes [51], and these mutants are readily recognized by HaloClass. *HaloClass showed particular strength with mutants involving lysines*. 23 of our 49 mutants involved a lysine (47%) and were responsible for no errors. The influence of lysines is evident: the evolutionary history of salt-tolerant proteins has generally selected against lysines [52]. Using one structural example, Pica et al. [5] hypothesized that lysines hinder salt tolerance because solvent interactions locking these longer side-chains in one conformation are more energetically costly. In line with this, Britton et al. [30] concluded that, in at least one case, when lysines remain in salt-tolerant proteins, they are highly ordered and less solvent-exposed compared to other proteins.

More broadly, we observed that HaloClass made no mistakes on the salt-tolerant DNA ligase but misclassified one of the mutants for the non-tolerant DNA ligase. This suggests that *HaloClass is marginally more performant on already salt-tolerant proteins*. Interested in understanding the significance of HaloClass confidences, we found that *larger HaloClass probability changes between wild-type and mutant sequences correlate with increased accuracy* (Supplementary Fig. 5).

Overall, HaloClass makes 3 mistakes out of 49 mutants for an accuracy of 94%, compared to 41% from Nath’s model. Nath’s model struggles on the mutation level with 21 of the 49 cases (43%) predicted to cause no change in salt tolerance.

To visualize wild-type and mutant structures, we generated AlphaFold 3 [13] models for the wild-type and an octuple-site mutant of the salt-tolerant DNA ligase N (Fig. 2). With an RMSD of just 0.6Å, we were impressed with HaloClass’s ability to differentiate between highly similar structures. These results on mutation level evaluation are especially surprising given that HaloClass was only trained on organism level annotations of salt tolerance. This suggests a strong ability for pLM embeddings to encode and derive relevant, task-specific information. Overall, our results on the mutation study suggest that, if applied carefully, HaloClass may have a stronger ability to support protein engineering campaigns than past approaches.

## Discussion

HaloClass is a new state-of-the-art algorithm for protein salt tolerance classification. By leveraging an ESM-2 language model for generating representations, HaloClass effectively learns to discriminate between a diverse range of proteins from distinct evolutionary and functional backgrounds. HaloClass was trained and evaluated on a larger dataset than past approaches enabling more robust generalizability.

On the Zhang dataset, HaloClass generalizes better than two algorithms that had already seen the sequences during training. On the Siglioccolo dataset, HaloClass becomes the first algorithm to make no ranking errors and makes only a single classification error on a 98-residue ferredoxin from *Nostoc sp.* PCC 7120. Structurally, this protein is in a tight fold stabilized by an iron-sulfur cluster, a motif that is hypothesized to increase protein stability [53, 54]. Recognizing this, we hypothesize two possible reasons for this HaloClass error. HaloClass might overestimate this pattern as contributing enough stability to achieve salt tolerance. Alternatively, this protein might genuinely bear enough stability to be salt-tolerant despite originating from a non-tolerant organism. However, without protein-level annotations, the exact cause is not attributable. In the future, new experimental data studying mutation-level salt tolerance can help elucidate HaloClass’s strengths and weaknesses.

In the mutation study, HaloClass demonstrates an ability to accurately predict mutational effects on salt tolerance. Broadly, HaloClass apparently learns the stability-related benefits of increased surface charge that mirror findings from Nath and other previous statistical analyses [7, 55]. We hypothesize that ESM-2 representations empower stronger performance due to its ability to encode long-distance amino acid interactions via the attention mechanism. In contrast, Zhang’s model includes no positional information about residues, while Nath’s model only accounts for dipeptides [6, 7]. Protein folding is a highly complicated process, medicated by the nuances of secondary and tertiary structure which necessarily involve long-range contacts [10, 12].

Future work should explore additional optimizations and techniques to improve performance on these techniques. Fine-tuning pLMs has been shown to increase performance for several downstream tasks, including thermostability prediction [56]. Related approaches, including codon language models [57], are worth future testing. Future projects could consider pLM representations with human-interpretable properties for more robust features. A more robust analysis of training dataset size and diversity is a meaningful direction for future exploration.

## Conclusion

We introduce HaloClass, an SVM model trained on ESM-2 representations that accurately classifies novel proteins based on salt tolerance. On the organism and structure level, we show that HaloClass generalizes better than past approaches on existing benchmarks and on our new test set. On the mutation level, we demonstrate that HaloClass can accurately distinguish changes in salt tolerance conferred by a variety of point mutants that alter both side-chain charge and length. These results suggest that HaloClass could have the ability to support protein engineering campaigns that are seeking to induce greater salt tolerance into existing enzymes. All code for HaloClass is available on GitHub, and a Jupyter notebook is available on Google Colab for model inference.

## Electronic Supplementary Material

Below is the link to the electronic supplementary material.

Supplementary Material 1

## Data Availability

All data and code are accessible on GitHub: https://github.com/kushnarang/haloclass-source, Google Colab: https://colab.research.google.com/drive/1UdzYqAxgN1ZXkrivfg_EUwk9Ryt0UBsx.

## Abbreviations

pLM: Protein language model
ESM: Evolutionary Scale Modeling
AUROC: Area under the receiver operating characteristic curve
MCC: Matthew’s correlation coefficient
RMSD: Root mean square deviation (in angstroms, Å)
