# Supplementary material for: HaloClass: Salt-Tolerant Protein Classification with Protein Language Models
Source: Protein J. 2024 Oct 21;43(6):1035–44. doi: 10.1007/s10930-024-10236-7 (PMC11543744; doi:10.1007/s10930-024-10236-7)
Supplement: Supplementary file 1 — Supplementary Material 1 [file 10930_2024_10236_MOESM1_ESM.docx]

# HaloClass: Salt-Tolerant Protein Classification with Protein Language Models

Kush Narang^1*^, Abhigyan Nath^2^, William Hemstrom^3^, Simon K. S. Chu^4^

* Corresponding author

^1^ College of Biological Sciences, University of California, Davis, United States

^2^ Department of Biochemistry, Pt. Jawahar Lal Nehru Memorial Medical College, India

^3^ Department of Biological Sciences, Purdue University, United States

^4^ Biophysics Graduate Program, University of California, Davis, United States

**Address correspondence to**:

Kush Narang

University of California, Davis

**E-mail**: knarang@ucdavis.edu

**ORCID**:<https://orcid.org/0009-0007-6726-3381>

**Supplementary Figure 1: Classification Model Selection**

We compared multiple different classification approaches to determine. For each model, 100 evaluation trials were conducted via bootstrapped sampling of the evaluation dataset. Either SVM or XGBoost was the highest performer on all four metrics. We selected SVM, because the fundamental algorithm is simpler than XGBoost. All models were evaluated on default settings, with probabilistic outputs enabled where that was required. KNN = K Nearest Neighbors, MLP = Multilayer Perceptron.


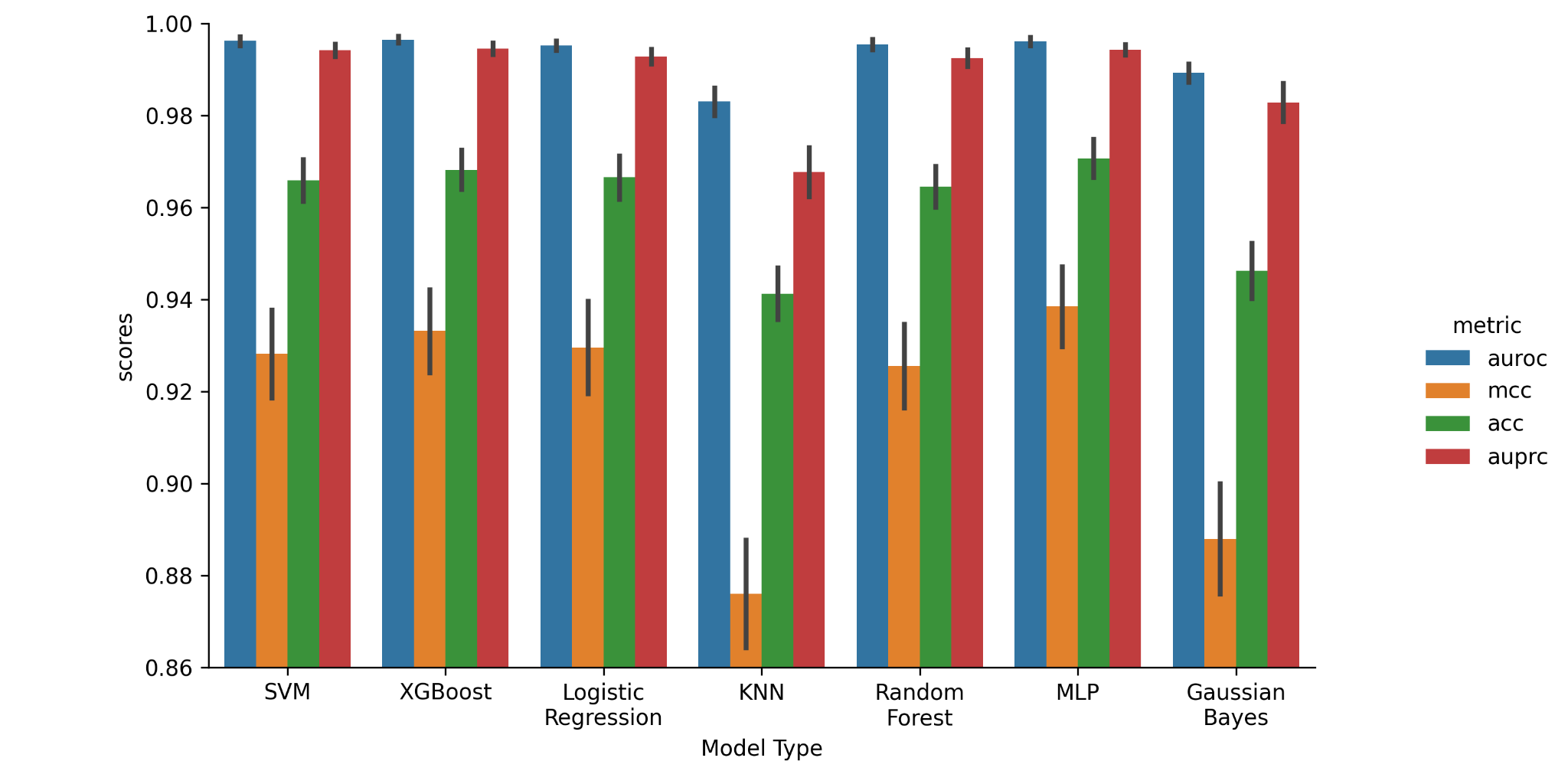


**Supplementary Figure 2: Language Model Selection**

We compared four different ESM-2 checkpoints as our feature extraction step to determine which would be optimal for the classification objective. We subset the evaluation dataset into a temporary train-eval split for this figure only to save on computational time. The checkpoints were the 8 million, 35 million, 150 million, and 650 million parameter versions of ESM-2. For each model, 100 evaluation trials were conducted via bootstrapped sampling. We selected the 150 million parameter model to optimize classification performance with reduced computational and storage demands.


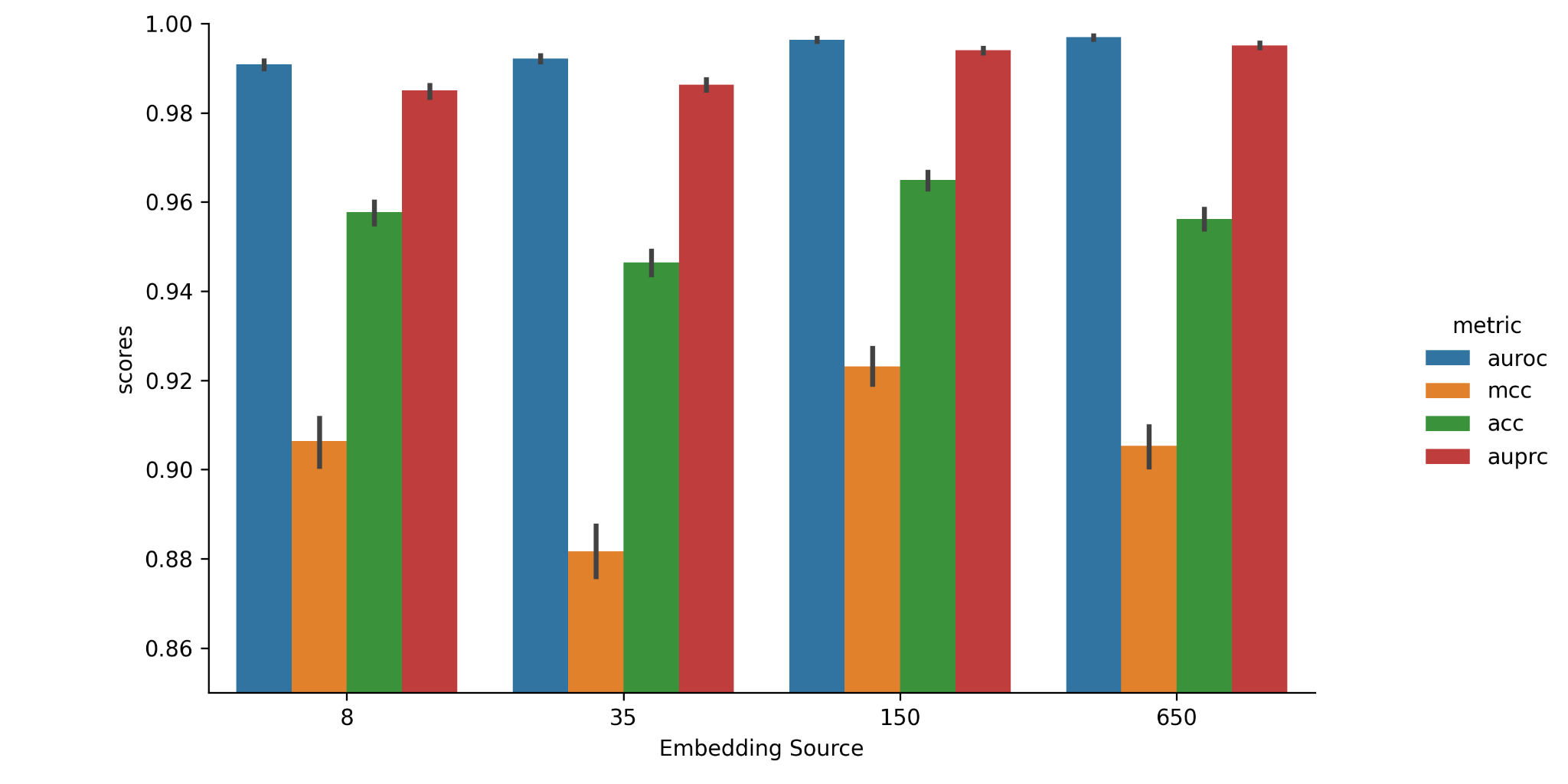


**Supplementary Figure 3: Kernel Selection**

We compared the four different kernel options available for SVM models in scikit-learn [citation needed]: linear, poly, rbf, and sigmoid. Here, linear, poly, and rbf performed similarly on all four metrics. For each model, 100 evaluation trials were conducted via bootstrapped sampling. We selected the linear kernel since it has the fastest training speeds, allowing for a more exhaustive hyperparameter search.


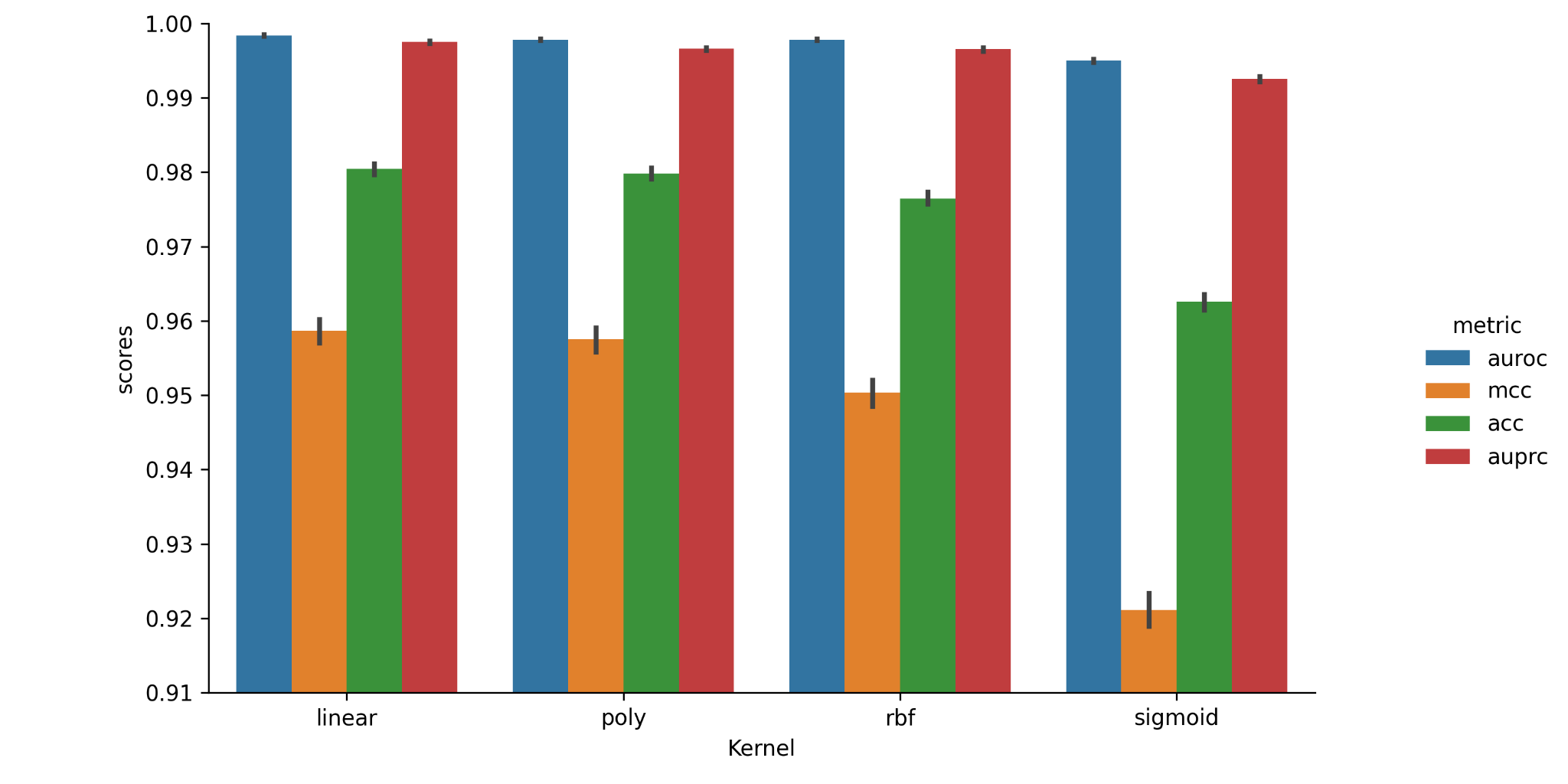


**Supplementary Figure 4: Hyperparameter Grid Search**

We compared SVM models that were trained with different hyperparameters for C and gamma. We tested all combinations of C = 0.1, 1.0, and 10, and gamma = 10^-6, 10^-5, 10^-4, 10^-3, 10^-2, 10^-1, and 1. The metric present is MCC. For each model, 100 evaluation trials were conducted via bootstrapped sampling. The highest performer was gamma = 1e-06 and C = 0.1, so we selected those parameters.


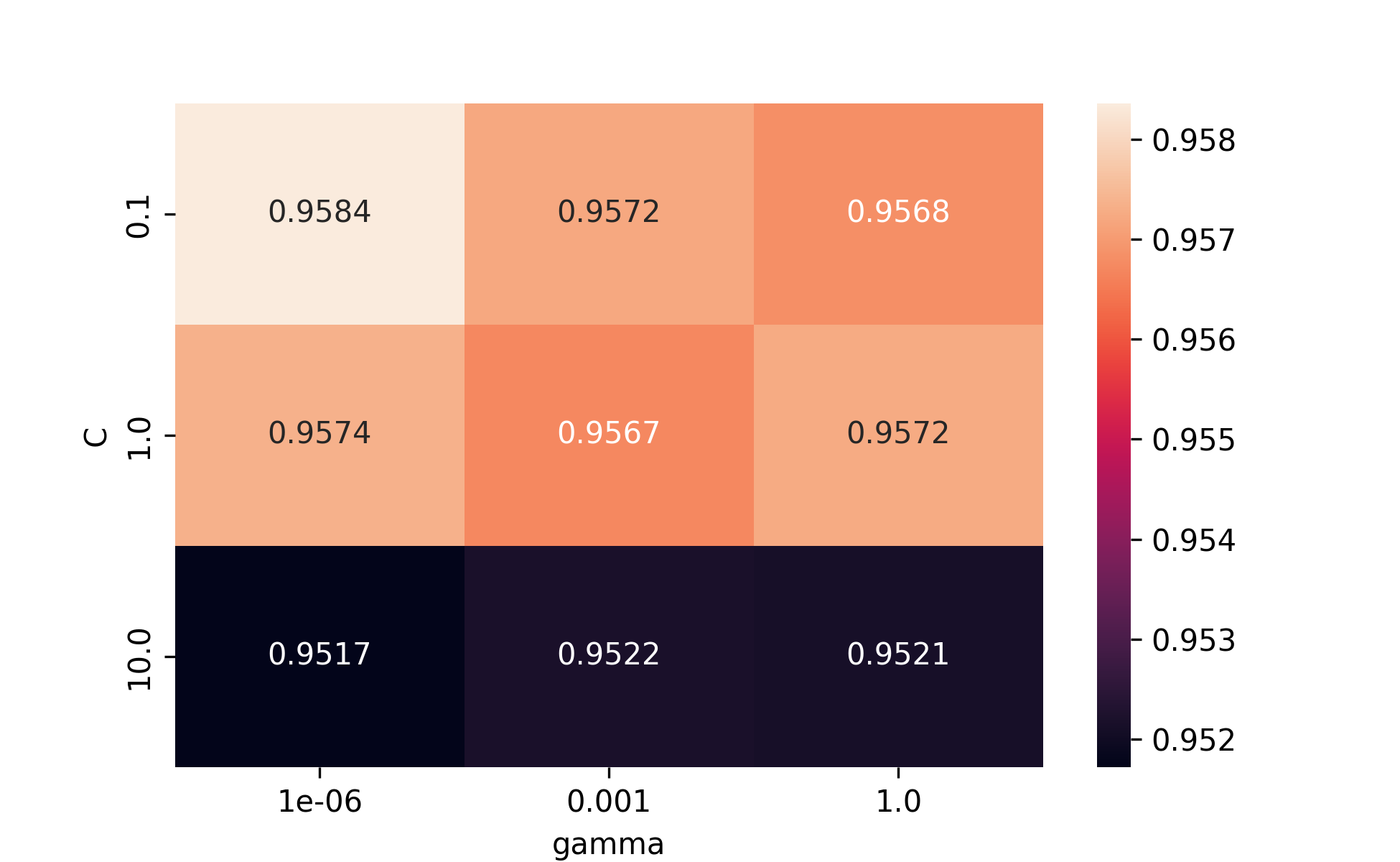


**Supplementary Figure 5: True and predicted changes in salt tolerance for mutants**

Density plot with true impact on salt tolerance versus predicted impact by HaloClass for the 49 mutants tested. Here, larger predicted deltas are correlated with great HaloClass accuracy.


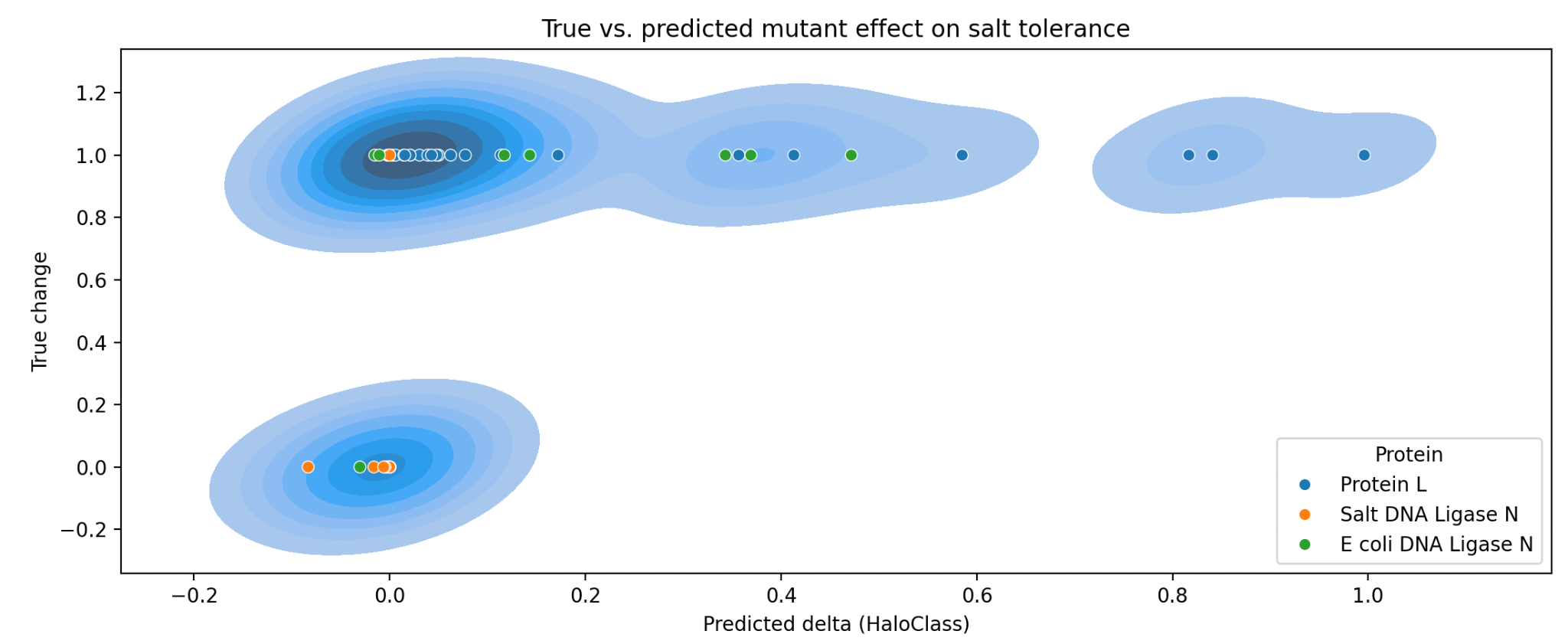


**Supplementary Table 1: Organisms and sequence counts in the new datasets**

| **Salt tolerant organisms** | **Non-tolerant organisms** |
| --- | --- |
| *Haloarcula marismortui* | *Aeropyrum pernix* |
| *Halobacterium salinarum* | *Escherichia coli* |
| *Halobacterium sp* | *Methanothrix thermoacetophila* |
| *Haloquadratum walsbyi* | *Shigella boydii* |
| *Salinibacter ruber DSM 13855* | *Thermococcus kodakarensis* |
|  | *Thermofilum pendens* |
|  | *Thermoplasma acidophilum* |
|  | *Thermotoga maritima* |
|  | *Pelobacter propionicus DSM 2379* |
|  |  |
| **15441 sequences** | **22920 sequences** |
| After 50% CD-HIT cutoff: | |
| **10424 sequences** | **17606 sequences** |
|  |  |
| **Final Datasets** | |
| **Training set** (90%): | 25227 sequences |
| **Testing set** (5%): | 1401 sequences |
| **Evaluation set** (5%): | 1402 sequences |

**Supplementary Table 2: Mean sequence identity similarities between training and evaluation datasets**

The mean sequence identity between the new test set and the training sets for HaloClass and the old approaches are 50% and 50.2%, respectively; these values indicate that the new test set serves as a fair test of generalizability for all the models. In contrast, evaluations on the Zhang dataset are expected to favor old approaches.

|  | **Training dataset** | |
| --- | --- | --- |
| **Evaluation dataset** | **Old approaches** (Zhang dataset) | **HaloClass** (New training set) |
| **Zhang dataset** | 100% | 71.7% |
| **Siglioccolo dataset** | 49.3% | 69.0% |
| **New test set** | 50.2% | 50% |

**Supplementary Table 3: Additional performance metrics for HaloClass on multiple datasets**

|  | **Precision** | **Recall** | **F1 score** |
| --- | --- | --- | --- |
| **Zhang dataset** | 1.00 | 0.88 | 0.93 |
| **New test set** | 0.99 | 0.97 | 0.98 |
| **Siglioccolo dataset** | 0.89 | 1.00 | 0.94 |

**Supplementary Table 4: Additional information about Siglioccolo dataset**

| **PDB** | **Source organism** | **Salt tolerant?** | **Function** |
| --- | --- | --- | --- |
| **1DOI** | Haloarcula marismortui | Yes | Ferredoxin |
| **1FXA** | Nostoc sp. PCC 7120 | No |  |
| **1TJO** | Halobacterium salinarum | Yes | DNA-protecting protein |
| **2VXX** | Synechococcus elongatus | No |  |
| **2B5W** | Haloferax mediterranei | Yes | Glucose dehydrogenase |
| **2CD9** | Saccharolobus solfataricus | No |  |
| **2CC6** | Halobacterium salinarum R1 | Yes | Dodecin |
| **2V18** | Thermus thermophilus HB8 | No |  |
| **1ITK** | Haloarcula marismortui | Yes | Catalase-peroxidase |
| **2FXG** | Burkholderia pseudomallei | No |  |
| **2AZ3** | Halobacterium salinarum | Yes | Nucleoside diphosphate kinase |
| **3B54** | Saccharomyces cerevisiae | No |  |
| **2J5K** | Haloarcula marismortui | Yes | Malate dehydrogenase |
| **1Y6J** | Acetivibrio thermocellus | No |  |
| **3IFV** | Haloferax volcanii | Yes | Proliferating cell nuclear antigen |
| **1RWZ** | Archaeoglobus fulgidus | No |  |
